# Hematopoietic miR155 Deficiency Enhances Atherosclerosis and Decreases Plaque Stability in Hyperlipidemic Mice

**DOI:** 10.1371/journal.pone.0035877

**Published:** 2012-04-25

**Authors:** Marjo M. P. C. Donners, Ine M. J. Wolfs, Lauran J. Stöger, Emiel P. C. van der Vorst, Chantal C. H. Pöttgens, Stephane Heymans, Blanche Schroen, Marion J. J. Gijbels, Menno P. J. de Winther

**Affiliations:** 1 Department of Molecular Genetics, Cardiovascular Research Institute Maastricht, Maastricht University, Maastricht, The Netherlands; 2 Department of Pathology, Cardiovascular Research Institute Maastricht, Maastricht University, Maastricht, The Netherlands; 3 Department of Cardiology, Cardiovascular Research Institute Maastricht, Maastricht University, Maastricht, The Netherlands; 4 Department of Medical Biochemistry, Academic Medical Center Amsterdam, Amsterdam, The Netherlands; Institut National de la Santé et de la Recherche Médicale, France

## Abstract

microRNA-155 (miR155) is a central regulator of immune responses that is induced by inflammatory mediators. Although miR155 is considered to be a pro-inflammatory microRNA, *in vitro* reports show anti-inflammatory effects in lipid-loaded cells. In this study we examined the role of miR155 in atherosclerosis *in vivo* using bone marrow transplantation from miR155 deficient or wildtype mice to hyperlipidemic mice. Hematopoietic deficiency of miR155 enhanced atherosclerotic plaque development and decreased plaque stability, as evidenced by increased myeloid inflammatory cell recruitment to the plaque. The increased inflammatory state was mirrored by a decrease in circulating CD4^+^CD25^+^FoxP3^+^ regulatory T cells, and an increase in granulocytes (CD11b^+^Ly6G^+^) in blood of miR155^−/−^ transplanted mice. Moreover, we show for the first time a crucial role of miR155 in monocyte subset differentiation, since hematopoietic deficiency of miR155 increases the ‘inflammatory’ monocyte subset (CD11b^+^Ly6G^−^Ly6C^hi^) and reduces ‘resident’ monocytes (CD11b^+^Ly6G^−^Ly6C^low^) in the circulation. Furthermore, cytokine production by resident peritoneal macrophages of miR155^−/−^ transplanted hyperlipidemic mice was skewed towards a more pro-inflammatory state since anti-inflammatory IL-10 production was reduced.

In conclusion, in this hyperlipidemic mouse model miR155 acts as an anti-inflammatory, atheroprotective microRNA. Additionally, besides a known role in lymphoid cell development, we show a crucial role of miR155 in myeloid lineage differentiation.

## Introduction

Atherosclerosis is a chronic inflammatory disease characterized by the accumulation of lipids and inflammatory cells in the vessel wall. Upon injury, monocytes are recruited to the vessel wall, where they differentiate into macrophages, scavenge lipids and become foam cells. Macrophages are a major source of inflammatory mediators in atherosclerotic lesions, contributing to the chronic nature of the inflammatory response. This chronic inflammatory response not only includes innate immune responses by myeloid cells but also adaptive immune responses by T- and B-cells.

MicroRNAs are small non-coding RNAs that negatively regulate expression of complementary genes by targeting messenger RNAs. Various microRNAs have been associated with inflammation and cardiovascular disease [1]. In this study we focus on microRNA-155 (miR155), a central regulator of immune responses that is highly expressed in activated immune cells [2] and various autoimmune inflammatory diseases [3]–[5]. miR155 expression in myeloid cells is induced by various inflammatory signals, including lipopolysaccharide (LPS), interferon-β and tumor necrosis factor [6]. It is considered to be a pro-inflammatory miRNA, mainly since one of its major targets is Suppressor of Cytokine Signaling (SOCS) 1, an endogenous inhibitor of inflammatory signaling [7]. Studies using miR155 deficient mice have shown defective B-and T-cell immunity, as well as impaired antigen-presenting functions of dendritic cells [3], [8], [9].

Besides inflammatory stimuli, exposure to oxidized low density lipoproteins (oxLDL) was shown to induce miR155 expression in human THP-1 macrophages [10]. oxLDL uptake and foam cell formation in these cells was increased when miR155 was inhibited and this was associated with upregulation of scavenger receptors [11], [12]. Interestingly, miR155 inhibition in these foam cells promoted NF-kB activity and the release of several pro-inflammatory cytokines [12], suggesting an anti-inflammatory rather than pro-inflammatory role of miR155 in atherosclerotic conditions. Most studies on the role of miR155 however have been performed *in vitro*, lacking the interaction and cross-talk with other cells. In this study we used miR155 deficient mice to study the exact role of miR155 in atherosclerosis development and plaque stability. We show that hematopoietic miR155 deficiency in hyperlipidemic mice increases atherosclerotic plaque burden and augments the inflammatory state in these mice.

## Results

### Hematopoietic deficiency of miR155 leads to larger, more advanced atherosclerotic lesions in LDLR^−/−^ mice

**Figure 1:**
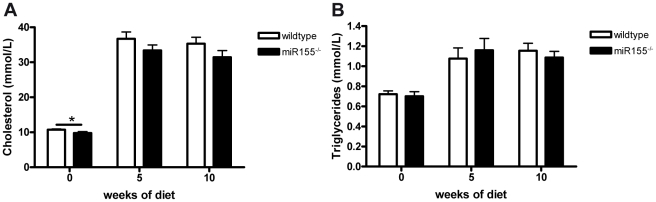
Plasma lipid levels of mice transplanted with either miR155^−/−^ or wildtype bone marrow. Cholesterol (A) and Triglyceride (B) levels measured before the start of HC diet (0 weeks of diet), after 5 weeks of diet and upon sacrifice of the mice (10 weeks of diet). * p<0.05, n = 20/group.

Considering the key role of miR155 in regulating inflammation and immunity and the chronic inflammatory nature of atherosclerotic plaques, we investigated the effect of hematopoietic absence of miR155 on atherosclerosis development. Therefore, we reconstituted bone marrow from miR155^−/−^ mice or their wildtype controls to lethally irradiated LDLR^−/−^ mice. Five weeks after reconstitution, mice were placed on a high cholesterol (HC) diet for 10 weeks after which atherosclerosis development was assessed in the aortic root. Overall chimerism determination showed similar (>95%) repopulation for both genotypes and no differences between groups were found in body weight (21.7±0.4 g for wildtype vs 21.7±0.3 g for miR155^−/−^ transplanted mice). Although plasma cholesterol levels at the start of the diet were slightly reduced in the miR155−/− transplanted mice (10.73±0.23 mmol/L for wildtype vs 9.79±0.38 mmol/L for miR155^−/−^, p = 0.04), no significant differences were found in either plasma cholesterol or triglyceride levels after HC diet feeding (figure 1). The slight reduction in plasma cholesterol before HC diet feeding was found in all lipoprotein fractions (i.e. VLDL, LDL and HDL, Figure S1A), indicating no shift in lipoprotein distribution between genotypes. No differences in cholesterol in these fractions were found after HC diet feeding for 10 weeks (Figure S1B).

**Figure 2:**
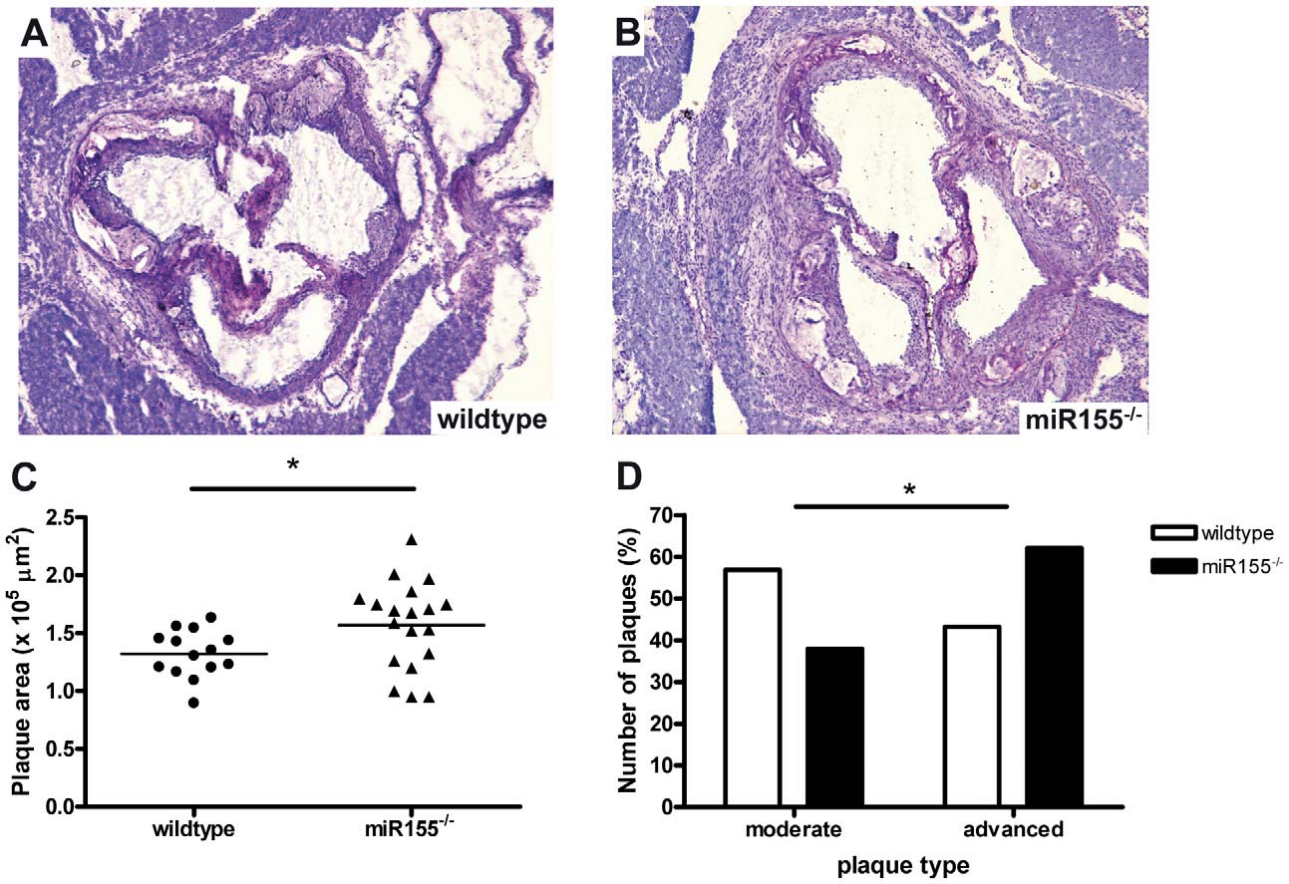
Hematopoietic deficiency of miR155 promotes atherosclerotic lesion development in LDLR^−/−^ mice. Representative pictures of toluidine blue-stained sections of the aortic root of wildtype (A) and miR155^−/−^ (B) transplanted mice, magnification 40×. Hematopoietic miR155 deficiency increases plaque area (C) and promotes plaque progression towards more advanced lesions (D, Chi square test p<0.05). * p<0.05, n = 14 wildtypes and 19 miR155−/− mice.

Quantification of the atherosclerotic lesion area in the aortic root revealed a small, but significant 18% increase in lesion area in the miR155^−/−^ transplanted mice compared to wildtype transplanted animals (figure 2A–C, p = 0.04). Furthermore, plaque classification revealed that plaques in miR155^−/−^ transplanted mice were more advanced compared to wildtype transplanted mice (figure 2D, Chi-square test p = 0.046).

### Hematopoietic deficiency of miR155 leads to more inflammatory atherosclerotic lesions

**Figure 3:**
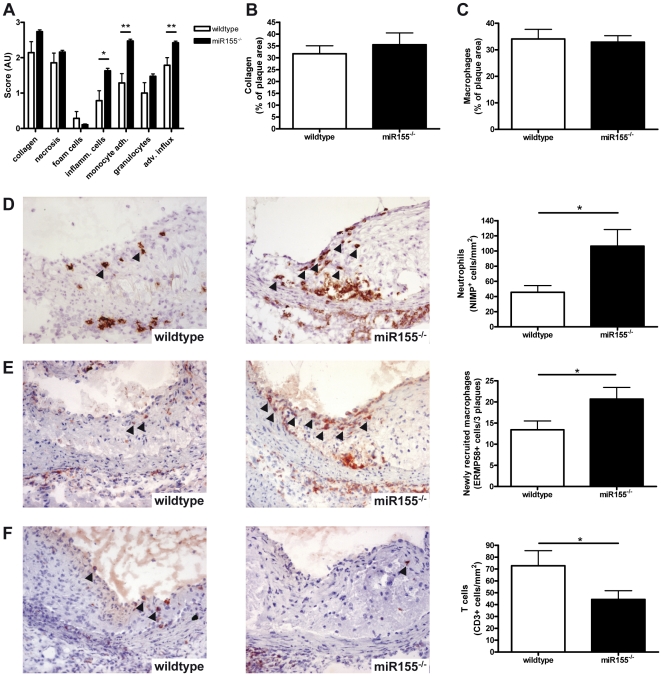
Hematopoietic deficiency of miR155 enhances inflammation in atherosclerotic lesions. (A) Pathological examination of collagen, necrosis, foam cells, inflammatory cells, monocyte adhesion to the plaque and adventitial influx. AU: arbitrary units. (B) collagen content stained by Sirius red staining, (C) Monocyte/macrophage area stained by moma-2, (D) Neutrophil numbers stained by NIMP, (E) Newly recruited macrophages identified by ERMP58 and (F) T cell numbers stained by KT-3 antibody (directed against CD3). Arrowheads indicate positively stained cells. *p<0.05, n = 14 wildtypes and 19 miR155−/− mice.

Atherosclerotic lesions were further examined by routine pathological examination for their composition, i.e. collagen content, necrosis, foam cell content, amount of inflammatory cells. Whereas no differences were found in collagen, apoptosis, necrosis or foam cell content, the number of inflammatory cells within the atherosclerotic lesion, adhering to the plaque or present in the adventitia was found to be increased (Figure 3A–C and Figure S2). Immunohistochemical quantifications revealed that this increase in inflammatory cells was mainly attributable to an increase in neutrophils (Figure 3D, p<0.05) and macrophage recruitment (Figure 3E, p<0.05), whereas the number of T cells in miR155^−/−^ transplanted mice was decreased compared to controls (Figure 3F, p = 0.05). Although we did not find a difference in relative monocyte/macrophage content between groups (Figure 3C), we found a significant increase in the number of newly recruited macrophages (ERMP58^+^, Figure 3E, p<0.05) [13]. In wildtype transplanted mice newly recruited small macrophages were mainly found in moderate lesion types and recruitment decreased when plaques progressed to advanced lesions. In contrast, in miR155^−/−^ transplanted mice we found more recruitment of new macrophages in advanced lesions than in earlier stages of the disease (Figure S3, p = 0.06). This may imply that while in wildtype lesions progression by monocyte recruitment slows down and stabilizes at a certain stage, in the miR155^−/−^ transplanted mice advanced lesions continue to attract circulating monocytes.

Since miR155 has been implicated in *in vitro* oxLDL uptake by human THP-1 macrophages [11], [12], we investigated *in vivo* foam cell formation by Oil Red O lipid staining of peritoneal macrophages after HC diet feeding [14]. We did not find any difference in the number of peritoneal foam cells between miR155^−/−^ or wildtype transplanted mice (Fig. S4), which confirmed our findings in the atherosclerotic lesions, where pathological scoring showed that foam cell content was also similar in both groups (Figure 3A).

Together these data show that miR155 conveys atheroprotective effects by limiting leukocyte recruitment to the atherosclerotic lesions rather than acting as a pro-inflammatory microRNA in this hyperlipidemic milieu.

### Pro-inflammatory phenotype of miR155 deficient mice is reflected in circulating leukocytes and peritoneal cells

**Figure 4:**
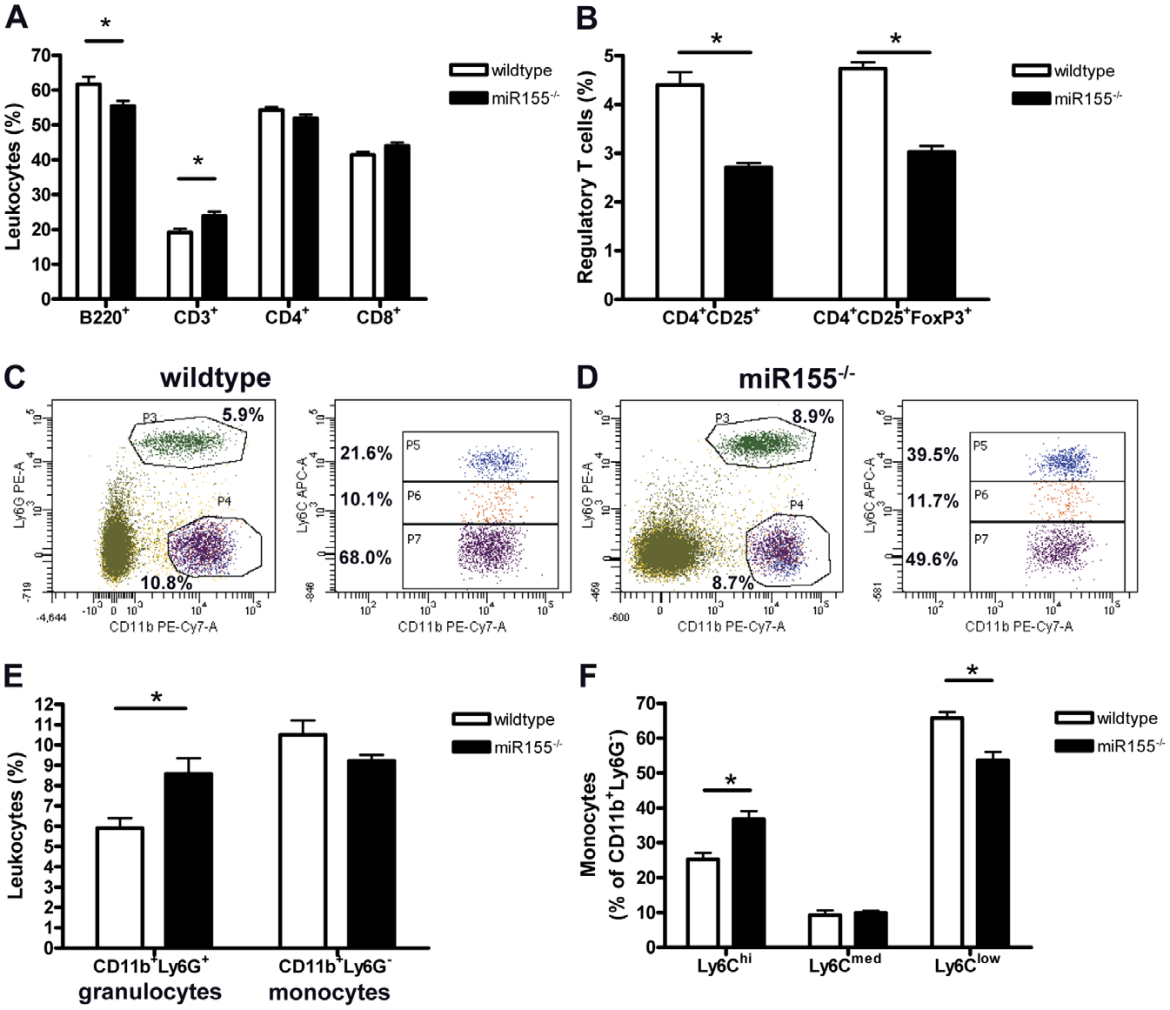
Pro-inflammatory blood leukocyte profile in hematopoietic miR155 deficient mice. (A) Blood lymphocytes profile. B220 for B cells, CD3 for T cells, CD4 T-helper cells and CD8 for cytotoxic T cells. (B) Regulatory T cells identified by CD4^+^CD25^+^ and CD25^+^FoxP3^+^ staining. Representative examples of circulating myeloid cells in wildtype transplanted (C) and miR155^−/−^ transplanted (D) mice. (E) Number of circulating granulocytes (CD11b^+^Ly6G^+^) and monocytes (CD11b^+^Ly6G^−^), subdivided in Ly6C^hi^ (inflammatory), Ly6C^low^ (resident) and Ly6C^med^ monocytes (F). *p<0.05, n = 10/group.

To determine whether the pro-inflammatory effects of hematopoietic deficiency of miR155 in the transplanted LDLR^−/−^ mice on HC diet are also reflected systemically, we performed FACS analyses on circulating leukocytes. In the lymphoid lineage, we found a small, but significant decrease in the number of B-cells (p = 0.02) and increase in CD3^+^ T cells (p = 0.01), although neither CD4^+^ T-helper cell nor CD8^+^ cytotoxic T cell subsets were affected by miR155 deficiency (Figure 4A). Interestingly, in agreement with findings of others under normolipidemic conditions [8], miR155 deficiency in hyperlipidemic mice decreased regulatory T cells (both CD4^+^CD25^+^ and CD25^+^FoxP3^+^ T_reg_ subsets) in the blood (Figure 4B, p<0.001).

**Figure 5:**
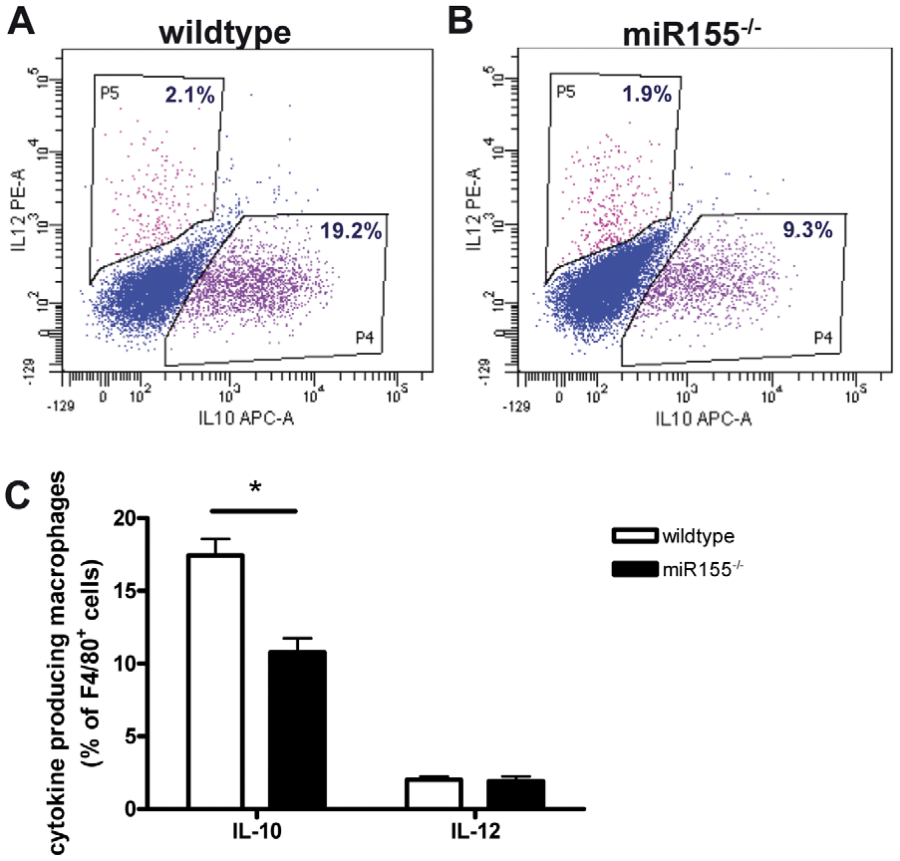
Hematopoietic miR155 deficiency decreases anti-inflammatory IL-10 production in peritoneal macrophages. IL-10 and IL-12 production by peritoneal macrophages of wildtype transplanted (A) and miR155^−/−^ transplanted (B) mice. Production of anti-inflammatory IL-10 in peritoneal macrophages of miR155^−/−^ transplanted mice is reduced (C). *p<0.05, n = 10/group.

Concerning the myeloid lineage, miR155 deficiency significantly increased the number of neutrophils (CD11b^+^Ly6G^+^, Figure 4C–E, p<0.01), reflecting the increased neutrophil content of the atherosclerotic lesions of miR155^−/−^ transplanted mice compared to wildtypes (Figure 3B). Remarkably, while circulating monocytes (CD11b^+^Ly6G^−^) was similar in both groups, miR155 deficiency in hyperlipidemic mice skewed the monocyte populations toward more pro-inflammatory (Ly6C^hi^) subsets (Figure 4C, D and F, p<0.001). To investigate whether miR155 is also important in the development of different macrophage subsets *in vivo*, we isolated resident peritoneal macrophages from the hyperlipidemic mice and analyzed inflammatory cytokine production after *in vitro* LPS stimulation by intracellular cytokine staining. Interestingly, although production of pro-inflammatory IL-12 was not affected, miR155 deficiency skewed macrophages towards a more pro-inflammatory phenotype by reducing the production of anti-inflammatory IL-10 (Figure 5, p<0.001).

### miR155 deficiency leads to increased pro-inflammatory macrophage responses in hyperlipidemic conditions only

**Figure 6:**
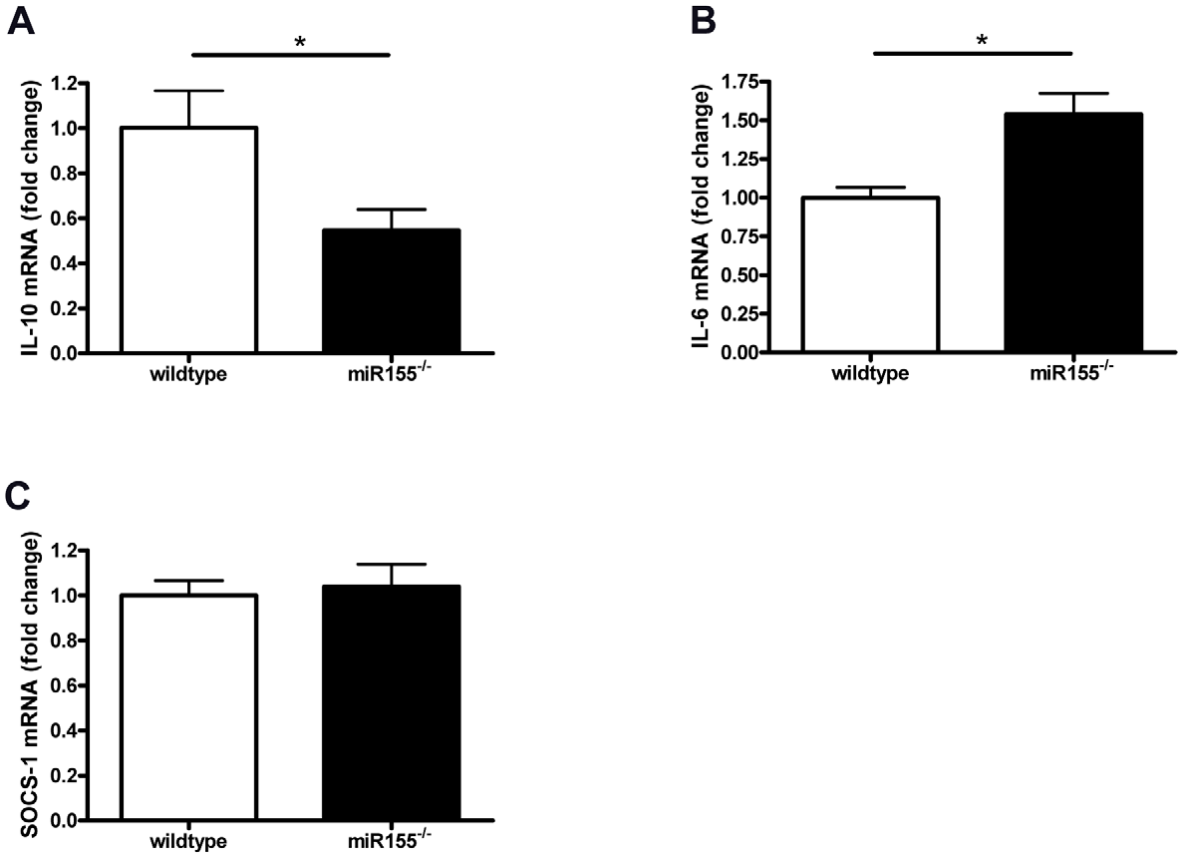
Pro-inflammatory effects of miR155 deficiency specifically in resident, hyperlipidemic peritoneal cells. mRNA was isolated from resident peritoneal cells obtained from wildtype or miR155^−/−^ transplanted mice after 10 weeks of HC diet feeding. miR155 deficiency decreases IL-10 mRNA expression (A), while increasing IL-6 mRNA expression (B) in these hyperlipidemic peritoneal cells. No differences were found between genotypes on SOCS-1 mRNA expression (C). *p<0.05, n = 9–10/group.

To elucidate the mechanisms behind these pro-inflammatory rather than the expected anti-inflammatory effects of miR155 deficiency, we isolated RNA directly from resident peritoneal cells from the hyperlipidemic wildtype and miR155^−/−^ transplanted mice and analyzed mRNA expression of SOCS-1, cytokines and NFkB-related genes. Whereas no differences were found between wildtypes and miR155^−/−^ cells in IL-12, TNF, MyD88 or IkBα (data not shown), we found a significant reduction in IL-10 expression in miR155^−/−^ transplanted mice (Figure 6A), confirming our results in resident, hyperlipidemic peritoneal macrophages (shown in Figure 5). Furthermore, we found an increase in pro-inflammatory IL-6 mRNA expression and no difference in SOCS-1 mRNA (Figure 6B and C, respectively).

We next investigated whether the pro-inflammatory effects of miR155 deficiency in our setting may be a direct result of the hyperlipidemia in HC diet fed LDLR^−/−^ mice. Therefore, we performed *in vitro* experiments with wildtype or miR155^−/−^ bone marrow derived macrophages that were loaded with oxLDL to convert them into foam cells. mRNA expression of SOCS-1, cytokines and NFkB-signaling genes were determined in oxLDL-loaded or non-lipid loaded macrophages after 3 hrs of LPS stimulation. As expected miR155 deficiency increased SOCS-1 expression in non-lipid loaded macrophages compared to wildtype macrophages (figure S5A). Interestingly, oxLDL loaded macrophages produce less SOCS-1 and miR155 deficiency in these foam cells fails to induce SOCS-1 expression. Whereas miR155 deficiency did not affect TNF, IL-6, IL-12, MyD88 or IkBα mRNA expression in either non-lipid loaded or oxLDL-loaded macrophages, IL-10 production was diminished in miR155^−/−^ foam cells, but not in non-lipid loaded macrophages (figure S5B). These data support our hypothesis that miR155 deficiency has more pro-inflammatory effects in hyperlipidemic, lipid loaded conditions rather than its anti-inflammatory effects under normal conditions.

## Discussion

This study is the first to show that miR155 has anti-inflammatory and atheroprotective effects in hyperlipidemic mice. Atherosclerotic lesions of miR155^−/−^ transplanted mice were not only larger, but also contained more inflammatory cells than those of control mice. The increased inflammation in atherosclerotic lesions was mirrored by increased numbers of circulating CD11b^+^Ly6G^+^ granulocytes and CD11b^+^Ly6C^hi^ inflammatory monocytes, while CD4^+^CD25^+^FoxP3^+^ T_reg_ cells were decreased in miR155^−/−^ transplanted mice. Similar decreases in T_reg_ cells were reported by others in miR155^−/−^ mice under normolipidemic conditions, although T_reg_ function appeared to be normal [8]. Moreover, production of the anti-inflammatory cytokine IL-10 was reduced in peritoneal macrophages isolated from hyperlipidemic miR155^−/−^ mice as well as in bone marrow derived miR155^−/−^ macrophages that were loaded with oxLDL *in vitro*. Moreover, miR155 deficiency leads to an increase in SOCS-1 expression in non-lipid loaded macrophages, but not in foam cells.

Although miR155 is generally considered to be a pro-inflammatory microRNA, by suppressing SOCS1, this *in vivo* study strongly indicates an opposite role in hyperlipidemic mice. Indeed, in unstimulated resident peritoneal cells from the *in vivo* bone marrow transplantation experiment we found no increase in SOCS-1 expression in miR155^−/−^ transplanted animals compared to controls. Furthermore, in these cells we found a reduction in IL-10 mRNA expression, in line with the reduced IL-10 protein expression in peritoneal macrophages from this *in vivo* experiment after *in vitro* LPS stimulation. Direct evidence supporting our hypothesis that in hyperlipidemia miR155 exerts opposite effects were demonstrated by our *in vitro* study with wildtype and miR155^−/−^ bone marrow derived macrophages that were either oxLDL-loaded or not. Here we showed that miR155 deficiency increases SOCS-1 expression in non-lipid loaded macrophages, but fails to do so in foam cells. Furthermore, IL-10 expression was only reduced in oxLDL-loaded macrophages deficient in miR155. Such anti-inflammatory function is in agreement with two *in vitro* studies showing that miR155 in lipid-loaded macrophages actually dampens their activation. Huang et al showed that antisense oligonucleotide silencing of endogenous miR155 in human THP-1 macrophages treated with oxLDL promoted the release of pro-inflammatory cytokines, i.e. IL-6, IL-8 and TNF-α [12]. This enhanced inflammatory response was accompanied by upregulation of MyD88 protein expression and NFkB translocation, a finding we could not confirm, at least not on mRNA levels. In line with the study of Huang et al and the current study, Chen et al. showed that miR155 inhibition also upregulated the adhesion molecules ICAM-1 and VCAM-1 in PMA-differentiated and oxLDL treated THP-1 cells [11]. Interestingly, both studies reported an increased scavenger receptor expression and increased oxLDL uptake by these cells. This finding however, could not be confirmed in our *in vivo* study, since no differences were found in lipid uptake by peritoneal macrophages or foam cell formation in the plaque.

The last decade, increasing evidence has shown large plasticity and heterogeneity in both monocytes and macrophage populations [15], [16]. In mice we mainly distinguish 2 monocyte subsets, i.e. CD11b^+^/Ly6C^hi^ ‘inflammatory’ monocytes that are rapidly recruited to sites of inflammation and give rise to inflammatory macrophages and dendritic cells, and CD11b^+^/Ly6C^low^ ‘resident’ or ‘patrolling’ monocytes that can enter the tissues under steady state conditions. Similarly we can distinguish various macrophage subsets, i.e. pro-inflammatory M1 macrophages and anti-inflammatory M2 macrophages [17]. M1 macrophages are induced by bacterial compounds or pro-inflammatory cytokines like IFNγ and have the capacity to increase and sustain the ongoing inflammatory response and to clear the system of bacterial, viral and fungal infections via production of inflammation-promoting mediators (e.g. IL-12). In view of their pro-inflammatory nature, Ly6C^hi^ inflammatory monocytes and M1 macrophages are considered to be pro-atherogenic. M2 macrophages, on the other hand, are induced by anti-inflammatory cytokines like IL4/13 and IL10 and have been shown to promote tissue repair and healing. M2 macrophages produce anti-inflammatory mediators such as IL-10, a cytokine well known to be atheroprotective [18]. To our knowledge this study is the first report of a role for miR155 in differentiation of monocyte and macrophage subsets. Besides an increase of the inflammatory Ly6C^hi^ monocyte subset in the circulation, miR155 deficiency also skewed macrophages in the peritoneal cavity towards a more pro-inflammatory phenotype, as their production of anti-inflammatory IL-10 was reduced. Interestingly, Ding *et al* showed the miR155 target SOCS1 to be an important inhibitor of IL-10 signaling [19]. Furthermore, recently a role of SOCS1 in macrophage polarization has been described *in vitro* [20]. In this study SOCS1 was shown not only to be important in controlling polarization towards the anti-inflammatory M2 macrophage phenotype, but also to regulate pro-inflammatory M1 macrophage responses e.g. by restricting IL-10 production. In line with these findings, our data may suggest a role for miR155 in macrophage polarization *in vivo*.

Together, our data show for the first time an atheroprotective role of miR155 and anti-inflammatory effects of miR155 *in vivo* in hyperlipidemic conditions. The increase of neutrophils and newly recruited macrophages in the atherosclerotic lesions was mirrored by a more pro-inflammatory blood leukocyte profile as evidenced by increased granulocytes and inflammatory monocytes, as well as a decrease in regulatory T-cells. Moreover, we not only showed an increase in circulating inflammatory monocytes but also decreased anti-inflammatory cytokine production by peritoneal macrophages, providing evidence for a role of miR155 in monocyte and macrophage subset differentiation.

## Methods

### Bone marrow transplantation

All animal experiments were approved by the DierExperimenten Commisie (DEC) of the Maastricht University (permit number 2009-168). Ten to twelve week old male miR155^−/−^ or wildtype C57Bl/6J mice were used as donor mice (n = 5 per group). Forty female LDLR^−/−^ mice (10–12 weeks) backcrossed onto a C57Bl6 background for more than 10 generations were obtained from in-house breeding. Bone marrow transplantations were performed as described elsewhere [21]. In short, LDLR^−/−^ mice were lethally irradiated with 6 Gy the day before and the day of transplantation. Mice were transplanted with 5×10^6^ bone marrow cells from wildtype or miR155^−/−^ mice. Five weeks after transplantation mice were put on a 16% fat, 0.15% cholesterol diet without cholate (Western type diet #4021.13, Hope Farms, The Netherlands). Before the onset of the high cholesterol diet, mice were fasted for 4 hours, after which blood was drawn from the tail vein for lipid analysis, and chimerism determination. Additional blood analysis was performed at 5 and 10 weeks of HC diet. The chimerism in transplanted mice was determined on blood DNA as described previously [22].

### Morphometry and immunohistochemistry murine tissues

After 10 weeks of Western-type diet feeding, mice were anesthetized and euthanized. Mouse hearts were dissected and snap frozen in OCT. Atherosclerosis development (lesion size and classification) was determined as described elsewhere [22]. Plaques were classified as early, moderate and advanced lesions. Early lesions consist of foam cells, but without formation of a necrotic core. Moderate lesions contain a fibrotic cap and often a necrotic core, but no macrophage infiltration into the media. Advanced lesions show (deep) infiltration of macrophages into the media and may contain elastic lamina degradation and more severe necrosis and fibrosis. Serial sections (7 µm) of the aortic root were cut and toluidine blue stained for morphometrical analysis and routine pathological examination for their composition, i.e. collagen content, necrosis, foam cell content, amount of inflammatory cells. TUNEL staining was used for detection of apoptotic cells. Sirius red staining was performed for the detection of collagen. Furthermore, sections were stained with Moma2 (a gift from G. Kraal) for the quantification of monocytes and macrophages, ERMP58 for early infiltrating macrophages (a gift from P. Leenen), KT3 for the detection of CD3^+^ T-cells and NIMP-1 for the detection of neutrophils (both in house cultured and purified).

### Lipid analysis

For size fractionation of lipoproteins, 60 µl of pooled plasma (three pools of 3 mice per group) was separated using an AKTA Basic chromotography system with a Superose 6PC 3.2/30 column (Amersham Biosciences, Roosendaal, The Netherlands). Total plasma cholesterol and triglyceride levels as well as cholesterol in lipoprotein fractions were determined using standard enzymatic kits according to manufacturer's protocols (Sigma-Aldrich, Zwijndrecht, the Netherlands).

### Flow cytometry analysis (FACS)

Blood leukocyte analysis was performed by FACS analysis. To discriminate between T-cells, B-cells, granulocytes and monocytes, cells were stained with either anti-CD3 (Ebioscience, clone 145-C11), CD45R/B220 (Ebioscience, clone RA3-6B2), Ly6G (BD, clone 1A8) or CD11b (BD, clone M1/70). Monocyte subsets were further delineated using Ly6C (Miltenyi, 1G7.G10) into inflammatory (Ly6C^hi^) and resident monocytes (Ly6C^−^). Furthermore, T-cell subsets were defined by CD4 (T-helper cells) (BD, clone RM4-5), CD8 (Cytotoxic T cells) (Ebiosciences, clone 53-6.7) and CD25 (Ebiosciencs, clone PC61.5) combined with FoxP3 (regulatory T cells) (Ebiosciences, clone FJK-16s).

Resident peritoneal macrophages were obtained by flushing the peritoneal cavity with ice-cold PBS followed by culturing in RPMI 1640 culture medium containing 10% FCS, penicillin (100 U/ml), streptomycin (100 ug/ml), and L-glutamine 2 mM (all GIBCO Invitrogen, Breda, The Netherlands). After overnight attachment, floating cells were removed while attached cells were activated by 10 ng/ml LPS in combination with GolgiSTOP (BD) for 6 hrs. Cells were subsequently collected and stained using F4/80-FITC (AbD serotec, clone CI:A31) and CD19-PERCPcy5.5 (Ebiosciences, clone 1D3) to discriminate between macrophages and B-cells respectively. To study intracellular IL-10 and IL-12 cytokine levels, cells were fixated using BD cytofix/cytoperm, permeabilized (BD perm/wash) and subsequently stained with IL-10-APC (Ebioscience, clone Jes5-16E3) and IL-12-PE (Ebioscience, clone C17.8). All flow cytometry measurements were performed by a FACS CANTO II (BD) followed by data analysis using FACSdiva software.

### *In vivo* peritoneal foam cell analysis

Peritoneal macrophages were collected as described above and stained for lipids using Oil Red O. The percentage of cells containing at least 2 lipid droplets was measured by counting at least 100 macrophages by light microscopy at 400× magnification.

### Bone marrow derived macrophage isolation and culture

Bone marrow cells were isolated from femurs and tibiae of either wildtype or miR155^−/−^ mice. Cells were cultured in RPMI-1640 (GIBCO Invitrogen, Breda, the Netherlands) with 10% heat-inactivated fetal calf serum (Bodinco B.V. Alkmaar, the Netherlands), penicillin (100 U/ml), streptomycin (100 µg/ml) and L-glutamine 2 mM (all GIBCO Invitrogen, Breda, the Netherlands) supplemented with 15% L929-conditioned medium (LCM) for 8–9 days to generate bone marrow-derived macrophages, as described previously [22].

Macrophages were seeded at 350000 cells per well in 24 wells plates and incubated 24 hrs with 0–25 µg/ml oxLDL (Intracel Resources, Frederick, Maryland, USA), followed by 3 hrs stimulation with 0–10 ng/ml LPS.

### Gene expression

RNA was isolated from resident peritoneal cells from the bone marrow transplantation experiment or from bone marrow derived macrophages with the High Pure RNA Isolation Kit (Roche, Basel, Switzerland). 500 ng total RNA was reverse transcribed using the iScript cDNA Synthesis Kit (BioRad, Veenendaal, the Netherlands). Quantitative PCR was performed using 10 ng cDNA, 300 nM of each primer, and SensiMix (Quantace-Bioline, London, UK) in a total volume of 20 µl. All gene expression levels were corrected for cyclophilin A, β-actin and GAPDH as housekeeping genes. Primer sequences are available upon request.

### Statistical analysis

Data are presented as mean ± the standard error of the mean (SEM). All statistical analyses were performed using the Prism program (GraphPad Software Inc, San Diego, CA). The statistical significance of differences was evaluated with the Student's t-test. Significance was accepted at the level of p<0.05.

## Supporting Information

Figure S1 **Representative examples of plasma VLDL, LDL and HDL cholesterol levels.** Cholesterol levels in all fractions were reduced in miR155^−/−^ transplanted mice before the start of HC diet feeding (A). No differences were found after 10 weeks of HC diet (B). n = 3 pools of 3 mice per group.
(TIF)

Figure S2 **Apoptosis is not affected by miR155 deficiency.** No differences were found in number of apoptotic cells in the atherosclerotic lesions as quantified by TUNEL staining. n = 11 wildtypes and 12 miR155−/− mice.
(TIF)

Figure S3 **Persisting monocyte recruitment in advanced lesions of miR155^−/−^ transplanted mice.** Increased number of newly recruited macrophages (ERMP58^+^, p = 0.06) in advanced atherosclerotic lesions, indicating persistence of monocyte recruitment, in miR155^−/−^ transplanted mice compared to wildtype transplanted mice. n = 20/group.
(TIF)

Figure S4 **Hematopoietic miR155 deficiency does not influence** ***in vivo*** **lipid uptake by peritoneal macrophages.** The percentage of Oil red O stained cells containing at least 2 lipid droplets was counted at 400× magnification. n = 10/group.
(TIF)

Figure S5 **Pro-inflammatory effects of miR155 deficiency specifically in foam cells.** Wildtype or miR155^−/−^ bone marrow derived macrophages (pools from 2 mice/group) were loaded with 0–25 µg/ml oxLDL for 24 hrs followed by 3 hrs LPS stimulation (10 ng/ml). miR155 deficiency leads to an increase in SOCS-1 mRNA expression in non-lipid loaded macrophages, but not in foam cells (A). IL-10 mRNA production is reduced in miR155^−/−^ foam cells, but not in non-lipid loaded macrophages (B). *p<0.05 miR155^−/−^ vs wildtype, treatments in triplicate.
(TIF)
