# Supplementary figures and images for: Hematopoietic miR155 Deficiency Enhances Atherosclerosis and Decreases Plaque Stability in Hyperlipidemic Mice
Source: PLoS One. 2012 Apr 25;7(4):e35877. doi: 10.1371/journal.pone.0035877 (PMC3338496; doi:10.1371/journal.pone.0035877)

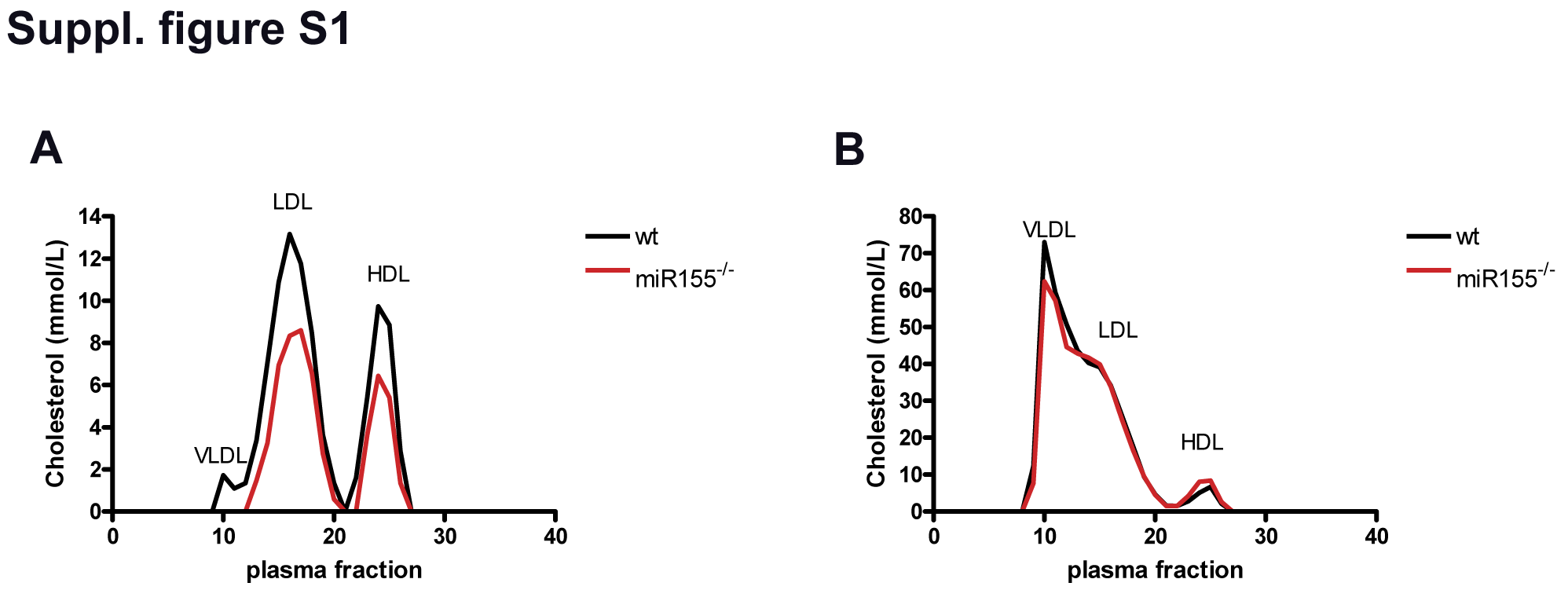

Supplement: Figure S1 — Representative examples of plasma VLDL, LDL and HDL cholesterol levels. Cholesterol levels in all fractions were reduced in miR155−/− transplanted mice before the start of HC diet feeding (A). No differences were found after 10 weeks of HC diet (B). n = 3 pools of 3 mice per group. (TIF) [file pone.0035877.s001.tif]

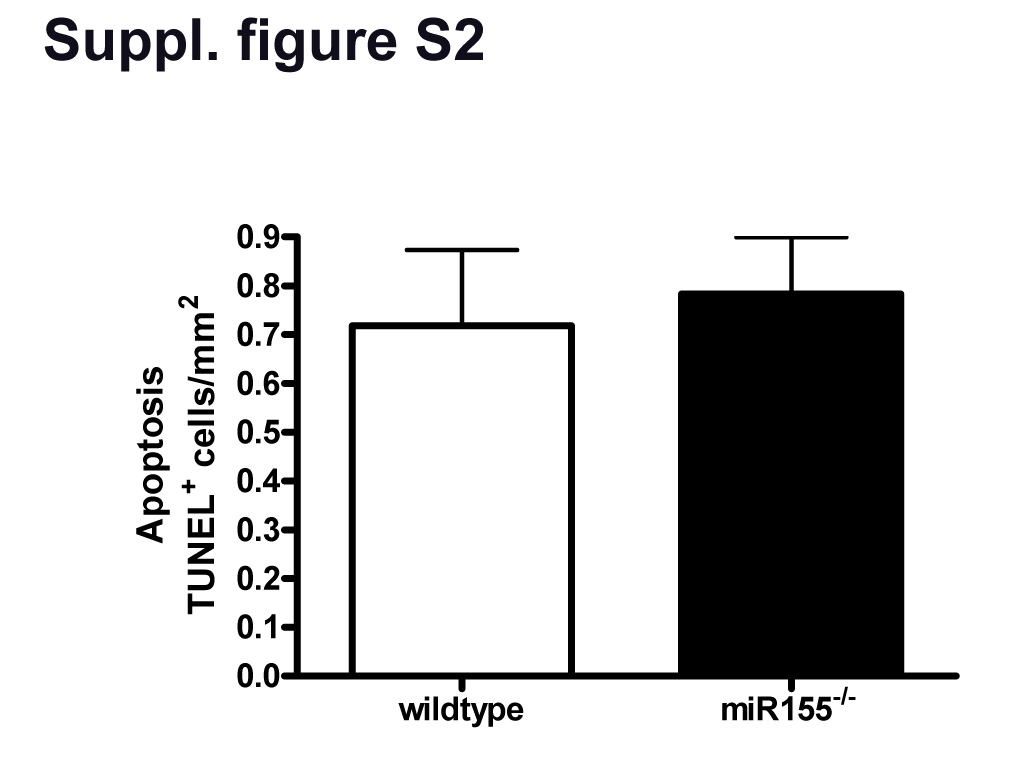

Supplement: Figure S2 — Apoptosis is not affected by miR155 deficiency. No differences were found in number of apoptotic cells in the atherosclerotic lesions as quantified by TUNEL staining. n = 11 wildtypes and 12 miR155−/− mice. (TIF) [file pone.0035877.s002.tif]

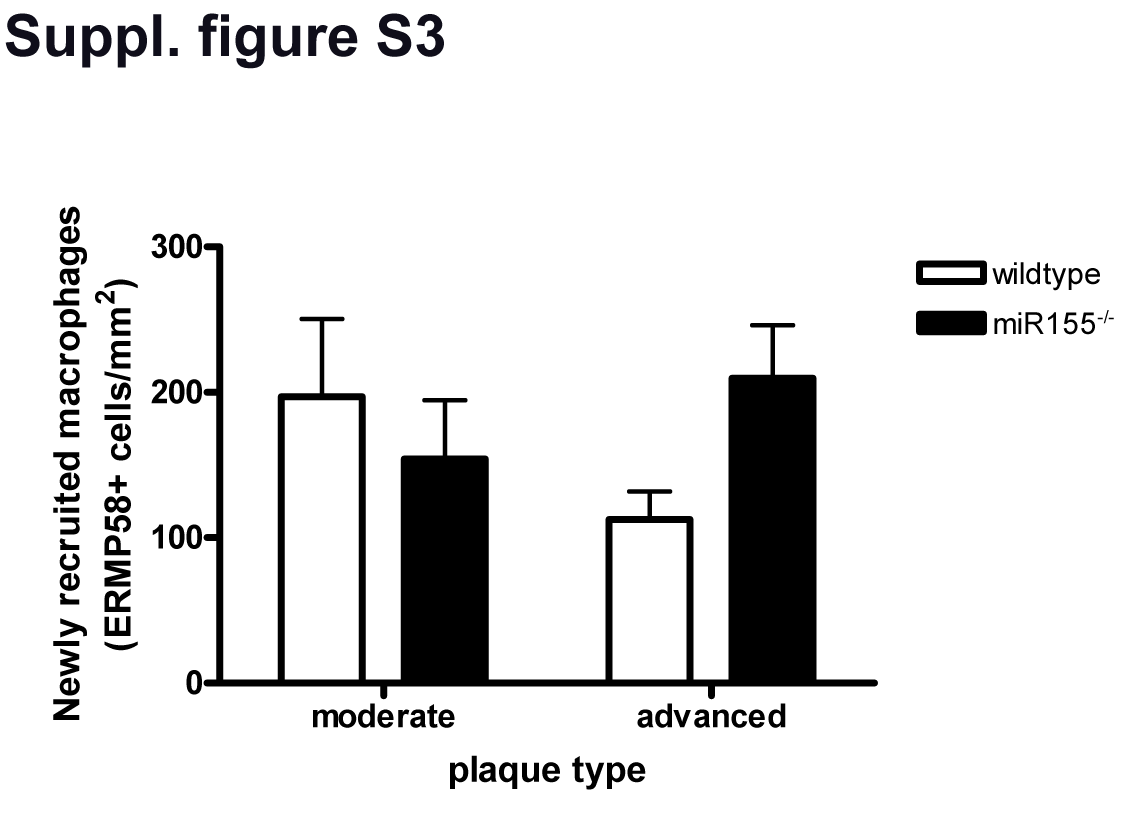

Supplement: Figure S3 — Persisting monocyte recruitment in advanced lesions of miR155−/− transplanted mice. Increased number of newly recruited macrophages (ERMP58+, p = 0.06) in advanced atherosclerotic lesions, indicating persistence of monocyte recruitment, in miR155−/− transplanted mice compared to wildtype transplanted mice. n = 20/group. (TIF) [file pone.0035877.s003.tif]

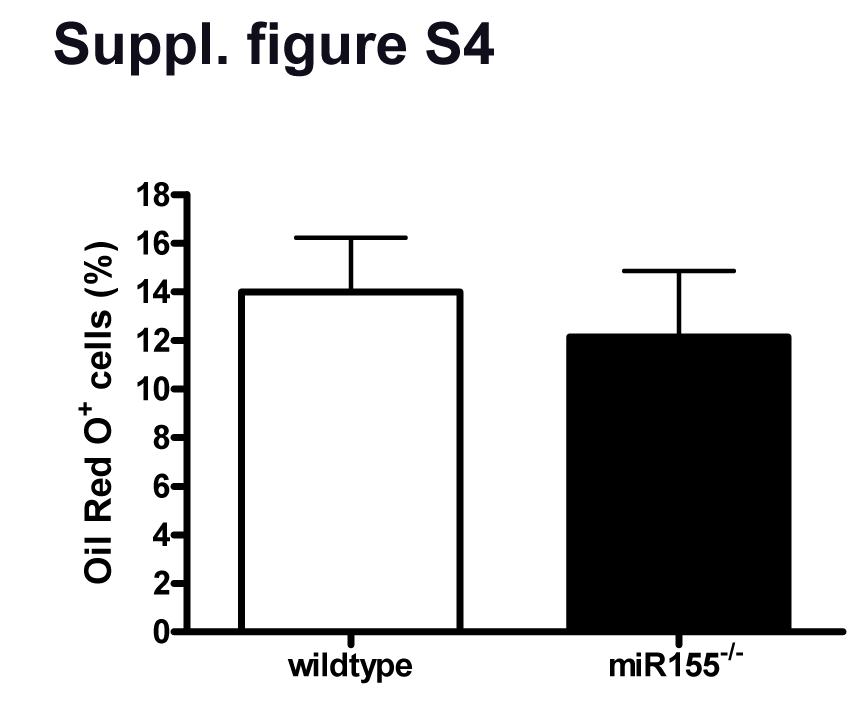

Supplement: Figure S4 — Hematopoietic miR155 deficiency does not influence in vivo lipid uptake by peritoneal macrophages. The percentage of Oil red O stained cells containing at least 2 lipid droplets was counted at 400× magnification. n = 10/group. (TIF) [file pone.0035877.s004.tif]

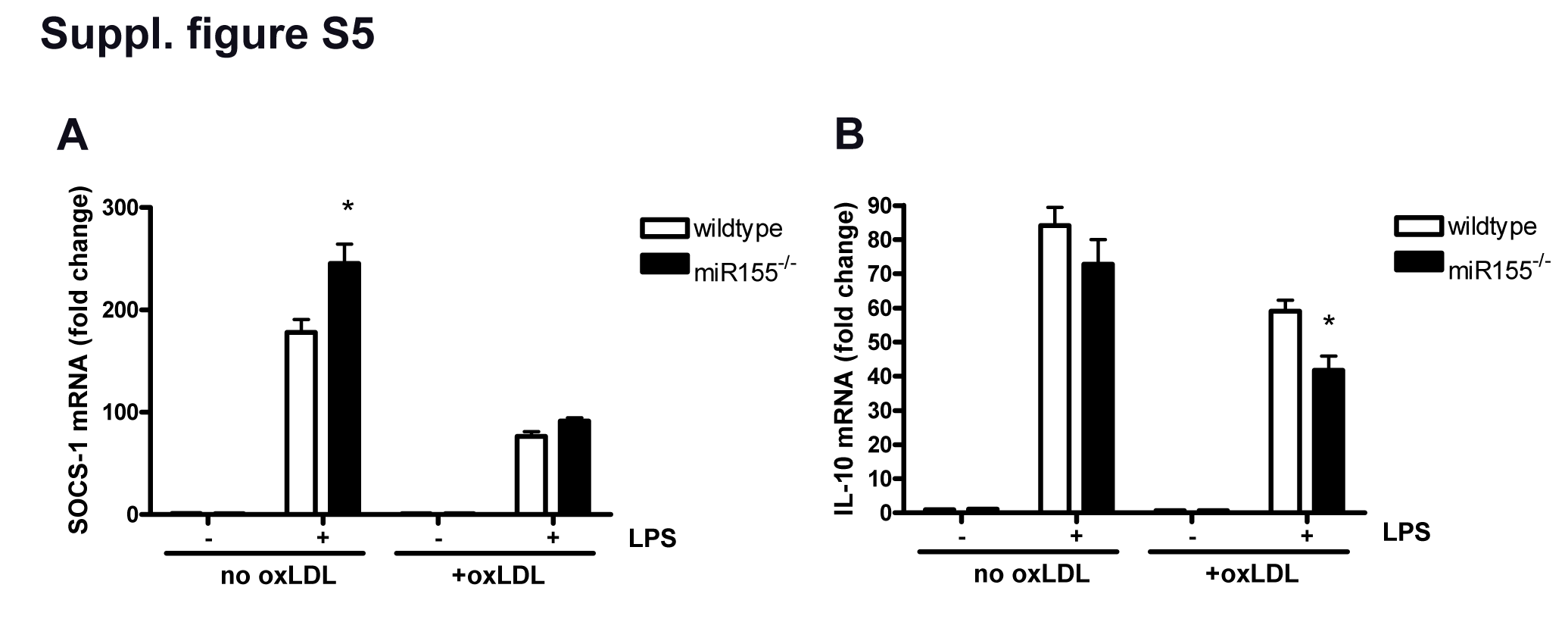

Supplement: Figure S5 — Pro-inflammatory effects of miR155 deficiency specifically in foam cells. Wildtype or miR155−/− bone marrow derived macrophages (pools from 2 mice/group) were loaded with 0–25 µg/ml oxLDL for 24 hrs followed by 3 hrs LPS stimulation (10 ng/ml). miR155 deficiency leads to an increase in SOCS-1 mRNA expression in non-lipid loaded macrophages, but not in foam cells (A). IL-10 mRNA production is reduced in miR155−/− foam cells, but not in non-lipid loaded macrophages (B). *p<0.05 miR155−/− vs wildtype, treatments in triplicate. (TIF) [file pone.0035877.s005.tif]
